# Functional characteristics of lactobacilli from traditional Bulgarian fermented milk products

**DOI:** 10.3906/biy-1808-34

**Published:** 2019-04-05

**Authors:** Veronica NEMSKA, Petya LOGAR, Tanya RASHEVA, Zdravka SHOLEVA, Nelly GEORGIEVA, Svetla DANOVA

**Affiliations:** 1 AQUACHIM JSC , Sofia , Bulgaria; 2 Department of Biotechnology, Faculty of Chemical and System Engineering, University of Chemical Technology and Metallurgy , Sofia , Bulgaria; 3 Department of General Microbiology, Stephan Angeloff Institute of Microbiology, Bulgarian Academy of Sciences , Sofia , Bulgaria

**Keywords:** Lactobacilli, probiotics, autoaggregation, adherence

## Abstract

After oral administration, probiotic lactobacilli meet a number of protection systems in the human body, such as exposure to gastric, pancreatic, and small intestinal juices. Overcoming these detrimental barriers allows living bacteria to adhere to the intestinal epithelium and permanently colonize the gastrointestinal tract (GIT), providing health benefits to the host. Based on this, the transit tolerance of 25 candidate probiotic lactobacilli from katak, yoghurt, and white-brined and yellow cheese to simulated bile and small intestinal juices of variable pH was investigated. To establish their resistance, in vitro model systems based on modified MRS media and a longer duration of action (up to 24 h of incubation) were designed. Six of the strains studied were found to show strain-specific survival capacity with low viability in conditions simulating stomach acidity and high resistance to bile and intestinal juices. In addition, the adherence capability (autoaggregation and hydrophobicity) of the strains was determined. Obtained results allowed to select Lactobacillus strains with high survival ratios while passing through the GIT and good adherence properties, which make them suitable for the development of new probiotics.

## 1. Introduction

Consumers today are quite well aware of the importance of healthy food for their health. For this reason, food containing probiotics, especially lactic acid products, is at the center of their attention due to their natural and safe origins. Probiotics are defined as live microorganisms, which have a positive effect on human health (Begley et al., 2006) . Among them, the representatives of the genus Lactobacillus are used predominantly in different probiotic products due to their greater resistance to low pH and better adaptation to milk and other substrates (Lee and Salminen, 2009) . They are proven to maintain the equilibrium of intestinal microflora, reduce the levels of blood cholesterol, stimulate mucosal immunity, and relieve lactose intolerance, diarrhea, constipation, and many other gastrointestinal disorders (Begley et al., 2006) .

In order to exert a positive effect, lactobacilli need to survive while passing through the different parts of the human gastrointestinal tract (GIT) and permanently colonize the colon. This determines the probiotic criterion for functionality as one of those with crucial importance. The main barriers influencing their survival are the pH changes in the GIT, bile salts in pancreatic juice, and different digestive enzymes in the small intestine. For this purpose, lactobacilli have developed different mechanisms by which they can reach the large intestine alive and in sufficient numbers. The acid tolerance is accomplished by maintaining intracellular pH, preservation of cell membrane functionality, and induction of stress response proteins. The release of bile acids/salts, hydrolysis of bile salts, and changes in the cell membrane and cell wall structure are among the most common mechanisms of bile salt resistance. In addition, proteins and fats are also found to play a protective role for probiotic bacteria. Adhesion to the epithelium of the colon is another crucial criterion for characterization of probiotics. It allows the retention of bacteria, which in turn maintain the microbial equilibrium and support the immune response (Bengoa et al., 2017) .

Overcoming the action of digestive juices from the pancreas, liver, and intestine, together with the following adhesion and colonization of the colon, are important selection criteria for new probiotics and their further industrial application. The aim of the present study was to evaluate the probiotic potential of 25 Lactobacillus strains from traditional Bulgarian dairy products.

## 2. Materials and methods

### 2.1. Microorganisms, media, and culture conditions

Twenty-five Lactobacillus strains from the laboratory collections of the University of Chemical Technology and Metallurgy and the Stephan Angeloff Institute of Microbiology (Bulgarian Academy of Sciences) were preselected for the present study. They were isolated from samples of home-made dairy products including katak, yoghurt, and white-brined and yellow cheese (Table 1). The pure cultures were stored at –20 °C in de Man, Rogosa, and Sharpe (MRS, HiMedia, India) broth, supplemented with 20% (v/v) glycerol. Before the assays, the strains were precultured twice in MRS broth at 37 °C under anaerobic conditions (Gas Pak 100 Anaerobic System, BD Bioscience, USA) for 24 h.

**Table 1:** Source and number of lactic acid bacteria (LAB) from traditional Bulgarian dairy products.

Fermented food	Raw material	LAB isolates
Katak	Sheep milk	Lactobacillus sp. S1 Lactobacillus rhamnosus S2 Lactobacillus plantarum S3 Lactobacillus fermentum S4
Yoghurt	Buffalo milk	Lactobacillus plantarum 1V, 2V, 3V, 7V, 8V Lactobacillus hamsteri 4V Lactobacillus sp. 5V, 6V Lactobacillus fermentum 9V
White-brined cheese	Sheep milk Buffalo and cow milk Goat milk Cow milk	Lactobacillus plantarum OC1, S6, S8, S9, S12 Lactobacillus lactis OC2 Lactobacillus plantarum S7 Lactobacillus plantarum BS32, BS41, S10 Lactobacillus salivarius КС2
Yellow cheese	Cow milk	Lactobacillus paracasei S11

### 2.2. In vitro tolerance to acidic pH

Exponential phase cells of each Lactobacillus strain were washed twice in phosphate buffered saline (PBS, pH 8.0), resuspended in modified MRS (mMRS) broth with different pH values (2.0, 3.0, 4.0, 5.0, and 6.0), and incubated at 37 °C for 24 h. The acid tolerance of lactobacilli was determined by cell counts calculated from the colonies on MRS agar after 24 h of incubation at 37 °C and expressed as a percentage of survival.

### 2.3. In vitro tolerance to bile salts

The strains were washed twice in PBS, resuspended in a simulated pancreatic juice (mMRS broth with different concentrations of bile salts (Oxgall, Merck, USA) - 0.3%, 0.5%, 1%, 1.5%, 2%, and 3% v/v) and incubated at 37 °C for 9 h. A sample in MRS (pH 6.5) without bile salts was used as a control probe. The resistance to bile salts was determined by plate count method as described previously and expressed as log 10 values of colony-forming units per ml (CFU ^–1^).

### 2.4. In vitro tolerance to small intestinal juices

Exponential phase cells of lactobacilli were washed twice in PBS and resuspended in a simulated intestinal juice with 0.1% trypsin (Sigma, USA) in PBS buffer (pH 8.0) and cultivated at 37 °C in an incubator (Binder, Germany). The tolerance to passage through the GIT was determined by measurements after 0, 3, 7, and 12 h of incubation. The staining method with trypan blue for distinguishing live from dead cells (Louis and Siegel, 2011) was used. The number of live/dead cells was determined by using a Vi-CELL XR Cell Viability Analyzer (Beckman Coulter, USA).

### 2.5. Hydrophobicity

The hydrophobicity of the bacterial cell surface was determined according to the method of Canzi et al. (2005) . Each Lactobacillus strain was inoculated into a biphasic system, containing 3 mL of PBS buffer (pH 6.4) and the same volume of n-hexadecane, and incubated at room temperature for 1 h. The absorption of the aqueous phase was measured at λ = 600 nm after 1 h and the results were expressed as a percentage of hydrophobicity.

### 2.6. Autoaggregation

The autoaggregation assay was determined according to the method of Bao et al. (2010) . Lactobacillus strains grown in MRS broth were harvested by centrifugation at 6000 rpm for 5 min, washed twice, and resuspended in PBS buffer (pH 6.0) to give an initial optical density of 0.25 at 600 nm. Absorbance was measured at different time points (0 and 20 h). The results were expressed as a percentage of autoaggregation.

### 2.7. Statistical analysis

All measurements were carried out in triplicate. The means were presented for averages of experiments ± standard deviation.

## 3. Results

Twenty-five Lactobacillus strains were subjected to a series of in vitro tests in order to evaluate their functional characteristics in the search for new probiotics with beneficial properties.

### 3.1. In vitro resistance to acidic pH

**Figure 1:**
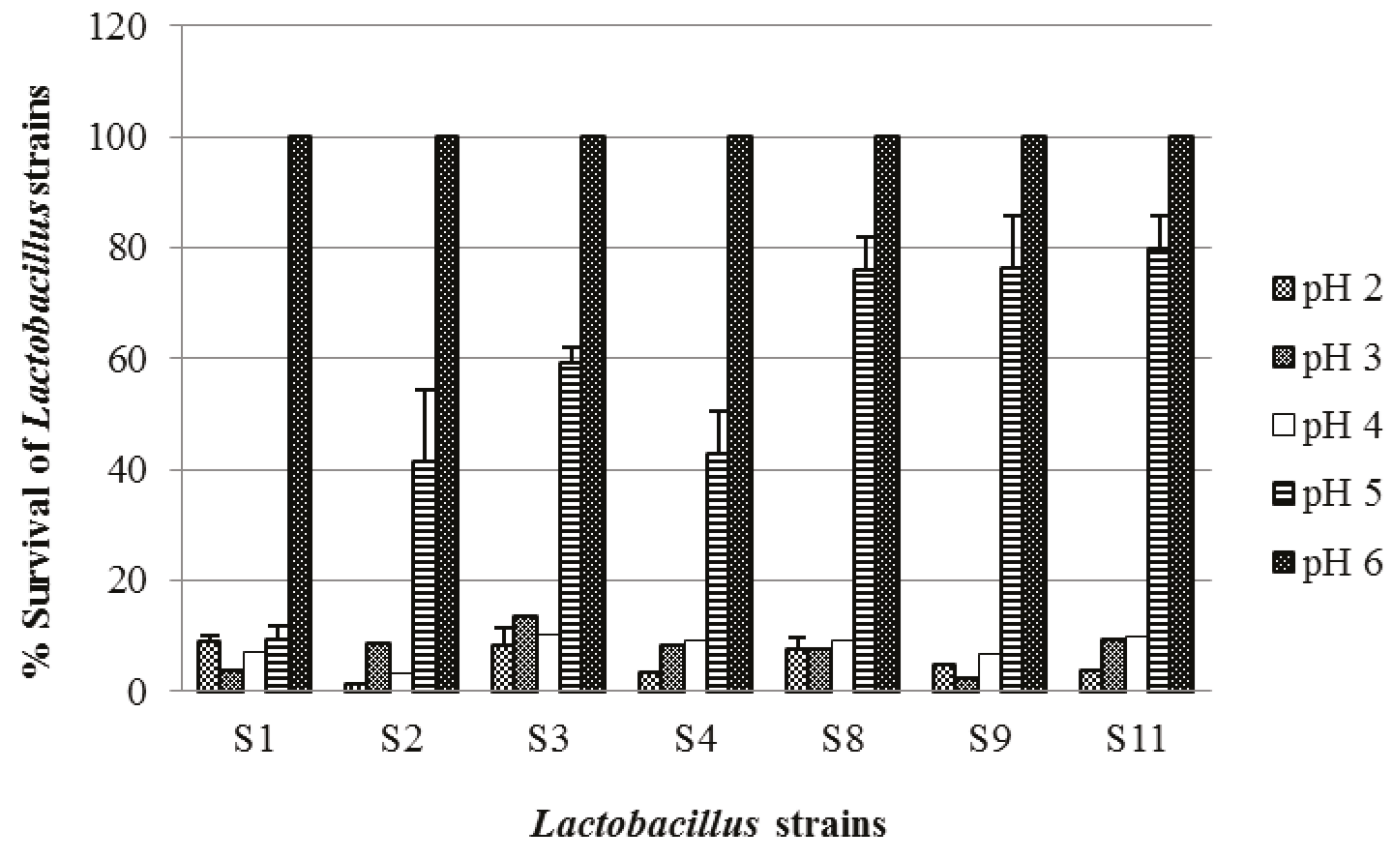
Survival (%) of tested lactobacilli at different pH values after 24 h of cultivation in mMRS broth. Data are expressed as means ± SD (n = 3). Error bars denote the standard deviations of three trials.

The survival of lactobacilli under conditions simulating the variable pH levels in the GIT is shown in Figure 1. All Lactobacillus strains grew well at pH 5.0, except strain Lactobacillus sp. S1. At lower pH (2.0 to 4.0), a significant reduction in growth rate was seen, with small variations between the strains.

### 3.2. In vitro resistance to bile salts

**Figure 2:**
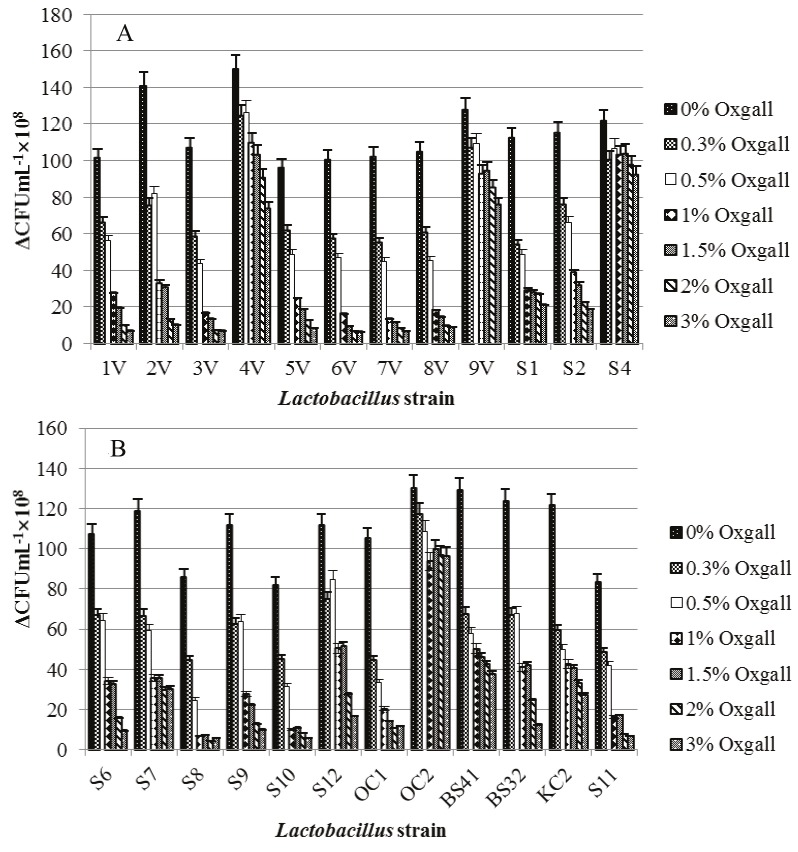
Survival (ΔCFU = (CFU initial – CFU after 9 h of treatment with bile salts) mL–1) of tested lactobacilli under simulated bile juice after 9 h of incubation in mMRS broth. Data are expressed as means ± SD (n = 3). Error bars denote the standard deviations of three trials.

The majority of strains showed similar survival counts (5 to 10 × 10^8^ CFU mL^–1^) during the first hour of incubation. Strains Lb. hamsteri 4V, Lb. fermentum S4, Lb. plantarum S12, and Lb. delbrueckii subsp. lactis OC2, isolated from yoghurt, katak, and white-brined cheese, respectively, are the only ones that showed higher vitality under these conditions (15 to 27 × 10^8^ CFU mL^–1^). By the third hour of incubation, visible differences between the strains were observed and strains Lb. plantarum BS32 and Lb. salivarius KС2 were added to the above-mentioned group of strains with high vitality. Results demonstrated that all the Lactobacillus strains showed high tolerance to 0.3% and 0.5% (w/v) bile salts (Figure 2). However, their viability gradually decreased with the gradual increase in bile salt concentration. After 5 h of incubation, the highest viability was accomplished for strains Lb. hamsteri 4V, Lb. fermentum 9V and S4, and Lb. delbrueckii subsp. lactis ОС2, which was preserved until the end of the experiment.

### 3.3. In vitro viability of Lactobacillus strains to small intestinal juices

**Figure 3:**
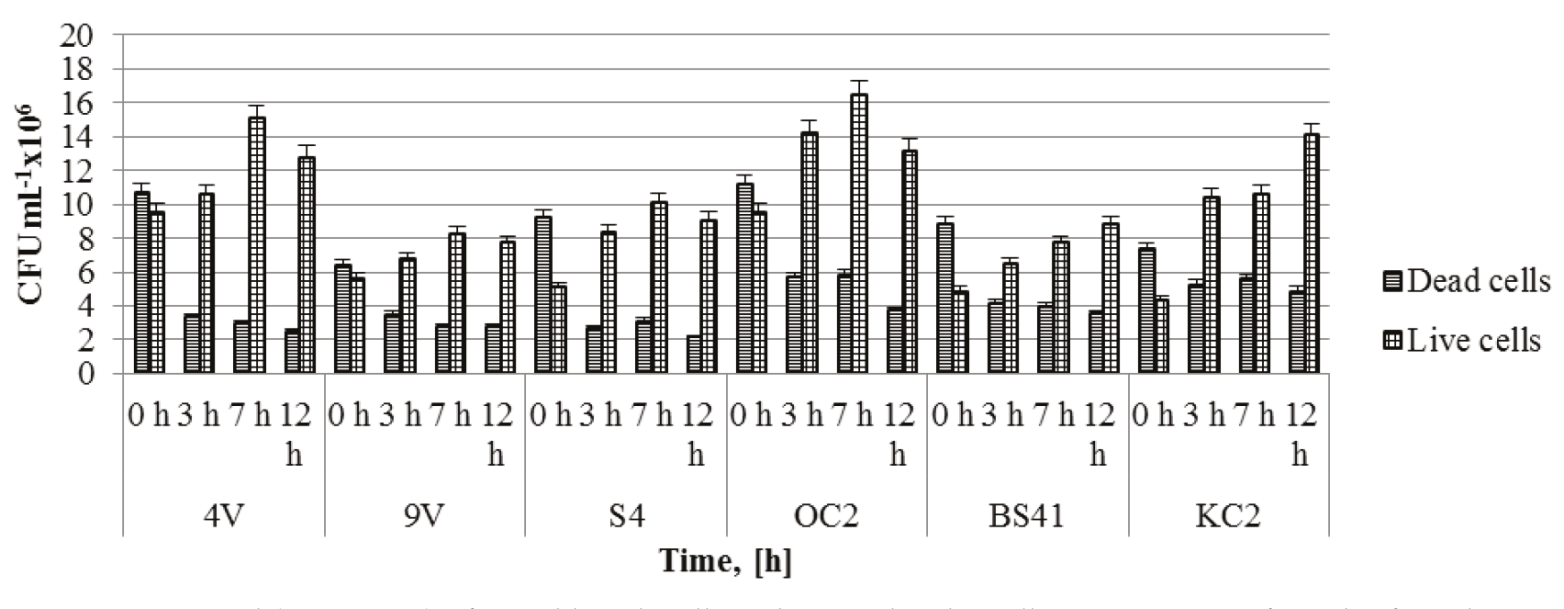
Survival (CFU mL–1) of tested lactobacilli under simulated small intestine juice after 3 h of incubation in mMRS medium. Data are expressed as means ± SD (n = 3). Error bars denote the standard deviations of three trials.

The simulation of physiological conditions in the small intestine was accomplished by incubating the lactobacilli that showed the highest probiotic potential under the action of bile salts with synthetic intestinal juice (mMRS broth with 0.1% (w/v) trypsin (Sigma, USA) and pH 8.0) for 12 h. The number of live and dead cells, respectively, was detected at 0, 3, 7, and 12 h of incubation by using a Vi-CELL XR Cell Viability Analyzer (Beckman Coulter, USA). The results were calculated and averaged for 1 mL of culture and are presented in Figure 3. Six of the studied lactobacilli could grow and develop under the simulated intestinal juice conditions. Lb. hamsteri 4V and Lb. delbrueckii subsp. lactis OC2 showed the highest viable counts (15.1 and 16.5 × 10^6^ CFU mL^–1^, respectively) after 7 h of incubation and the lowest were observed for Lb. fermentum 9V and Lb. plantarum BS41 (8.24 and 7.74 × 10^6^ CFU mL^–1^, respectively).

### 3.4. Autoaggregation and hydrophobicity

Adhesion to the intestinal epithelium and subsequent colonization of the GIT are basic criteria for the selection of probiotic strains. For this purpose, an in vitro determination of the microbial adhesion of 6 preselected Lactobacillus strains to nonpolar solvent n-hexadecane was carried out in accordance with Canzi et al. (2005) . In parallel, an analysis of their ability to autoaggregate was also made (Table 2).

**Table 2:** Aggregation and adhesion properties of Lactobacillus spp. in laboratory conditions.

Strain	Autoaggregation, [%]	Hydrophobicity, [%]
Lb. hamsteri 4V	2.86	6.34
Lb. fermentum 9V	9.88	0.4
Lb. fermentum S4	23.01	12.8
Lb. delbrueckii subsp. lactis OC2	10.1	7.32
Lb. plantarum BS41	16.17	10.17
Lb. salivarius KC2	18.47	4.94

The hydrophobicity of the lactobacilli ranged from 0.4% to 12.8%. Lb. fermentum 9V exhibited less than 1% retention capacity for n-hexadecane, which is a sign of low, almost absent, hydrophobicity, whereas Lb. fermentum S4 demonstrated the highest hydrophobicity at 12.8%. Among the strains studied, only Lb. fermentum S4 and Lb. plantarum BS41 exhibited hydrophobicity over 10%. At the same time, the autoaggregation between lactobacilli examined in this study varied from 2.86% (Lb. fermentum S4) to 23.01% (Lb. hamsteri 4V) (Table 2).

## 4. Discussion

The second important criterion in the selection of probiotic strains relates to their functional properties such as viability and transit tolerance under conditions simulating the passage through the human GIT and the ability to adhere to the intestinal mucosa.

Survival of LAB during the gastric passage depends on their ability to tolerate the action of released hydrochloric acid during digestion and has an impact on bacterial growth and functionality. The tolerance of lactobacilli to acidic medium is species- and strain-specific (Fontana et al., 2013) and it is determined by their ability to maintain a constant pH gradient between the pH of the medium and that of the cytoplasm. Good survival of the species Lb. plantarum at low pH was also observed by Yelnetty et al. (2014) . The relatively low percentage of survival in acid conditions (pH 2.0, 3.0, and 4.0) is probably due to the incubation period, which is 8 times longer than that of a normal passage through the GIT in vivo. In addition, the low pH causes damage to the microbial cell envelope, which is likely to increase the susceptibility to the action of bile salts and pancreatic enzymes in the duodenum (Elli et al., 2006) . This determines the in vitro resistance of the studied lactobacilli to bile salts as the second most important characteristic required for the recognition of a bacterial strain for a probiotic.

In the present study, all studied lactobacilli showed different survival abilities to varying concentrations of bile salts (0.3%, 0.5%, 1%, 1.5%, 2%, and 3% v/v, Oxgall, Merck, USA). Our results confirmed the findings of Bezkorovainy (2001) that bacterial survival depends not only on the concentration of bile salts but also on the time of the exposure to them. According to the classification of Ashraf and Smith (2016) , strains Lb. hamsteri 4V, Lb. fermentum 9V and S4, Lb. salivarius KC2, and Lb. delbrueckii subsp. lactis OC2 may be defined as bile-tolerant (survival >60% after 9 h of incubation). The results shown in Figure 3 also confirmed the reports of other authors concerning the species- and strain-specific nature of bile tolerance (Chateau et al., 1994; Gopal et al., 1996; Ziarno, 2007) .

In order to reach and colonize the colon and to express their probiotic effects, the candidate probiotic strains should stand the action of 0.7 L of intestinal juice with pH ~8.0, 0.5% (w/v) salt content, and pancreatic enzymes, synthesized daily in the proximal part of the small intestine. For this purpose, the potential of the 6 lactobacilli that showed the highest survival ability under the action of bile salts was tested for their viability and transit tolerance to simulated intestinal juices. After 7 h of incubation under these conditions, the strains under investigation did not show a significant difference in the growth parameters and the number of the live cells reported. With the exception of strains Lb. plantarum BS41 and Lb. salivarius KC2, the number of viable cells continued to increase until the end of the experiment (12 h). Findings revealed that the investigated Lactobacillus strains had great potential to withstand the passage through the small intestine.

As well as the other functional characteristics, hydrophobicity and autoaggregation are also strainspecific. All of the studied lactobacilli have low ability to adhere to the intestinal mucosal surfaces in accordance with the requirements for at least 85% hydrophobicity (Perez et al., 1990) . According to their autoaggregation potential, all Lactobacillus strains can be considered as strains with low (Lb. hamsteri 4V and Lb. fermentum 9V) or moderate autoaggregation capacity (all other strains) as determined by the classification of Wang et al. (2010) .

The present paper applies a combination of in vitro assays to access the beneficial properties and effectiveness of newly characterized lactobacilli from traditional dairy products including yoghurt, katak, and white-brined and yellow cheese. Strain-specific viability of lactobacilli in conditions simulating different parts of the GIT was estimated. In vitro tests are appropriate for successful preselection of new candidate probiotics. The high transit tolerance and the autoaggregation capacity of strains L. plantarum BS41 and L. fermentum S4 allow them to be selected as putative probiotics. Further characterization, however, is needed and is still in progress.
